# Intermartensitic Transformation and Enhanced Exchange Bias in Pd (Pt) -doped Ni-Mn-Sn alloys

**DOI:** 10.1038/srep25911

**Published:** 2016-05-12

**Authors:** S. Y. Dong, J. Y. Chen, Z. D. Han, Y. Fang, L. Zhang, C. L. Zhang, B. Qian, X. F. Jiang

**Affiliations:** 1 Jiangsu Laboratory of Advanced Functional Materials, Department of Physics, Changshu Institute of Technology, Changshu 215500, People’s Republic of China; 2 School of Materials Science and Engineering, China University of Mining & Technology, Xuzhou 221116, People’s Republic of China; 3 School of Science, Jiangnan University, Wuxi 214122, People’s Republic of China

## Abstract

In this work, we studied the phase transitions and exchange bias of Ni_50−x_Mn_36_Sn_14_T_x_ (T = Pd, Pt; x = 0, 1, 2, 3) alloys. An intermartensitic transition (IMT), not observed in Ni_50_Mn_36_Sn_14_ alloy, was induced by the proper application of negative chemical pressure by Pd(Pt) doping in Ni_50−x_Mn_36_Sn_14_T_x_ (T = Pd, Pt) alloys. IMT weakened and was suppressed with the increase of applied field; it also disappeared with further increase of Pd(Pt) content (x = 3 for Pd and x = 2 for Pt). Another striking result is that exchange bias effect, ascribed to the percolating ferromagnetic domains coexisting with spin glass phase, is notably enhanced by nonmagnetic Pd(Pt) addition. The increase of unidirectional anisotropy by the addition of Pd(Pt) impurities with strong spin-orbit coupling was explained by Dzyaloshinsky-Moriya interactions in spin glass phase.

Ni-Mn-X (X = In, Sn, Sb) ferromagnetic shape memory alloys (FSMAs), first reported by Sutou *et al.* in 2004^1^, have become an active field of research because of the great richness of physics as well as their potential applications in magnetic refrigerator, sensor, and actuator. The strong magnetostructual coupling in the vicinity of the martensitic transformation (MT) results in multifunctional properties such as large magnetocaloric effect^2^,^3^,^4^,^5^,^6^, barocaloric effect^7^, magnetoresistance, and metamagnetic shape memory effect^8^,^9^,^10^ in these alloys. A good control of phase transition and magnetism of Ni-Mn-X alloys is of great importance to improve their functional properties. It has been reported that MT is strongly dependent on the valence electron concentration (e/a)^11^, pressure^12^,^13^, chemical order^14^,^15^, and crystalline size^16^. In the study of the pressure effect, the application of physical or chemical pressure in Ni-Mn-In and Ni-Mn-Sn alloys has been found to shift the transformation temperature toward higher temperature associated with the decrease of cell volume^12^,^17^,^18^. Recently, an intermartensitic transformation (IMT) and enhanced MT temperature were induced in high pressure annealed Ni-Co-Mn-Sn alloy, producing improved magnetocaloric effect^19^. These results suggest the great impact of pressure on the phase transitions in Ni-Mn-X alloys. Furthermore, both experimental and theoretical investigations show the magnetic properties of Ni-Mn-X Heusler alloys are extremely sensitive to hydrostatic pressure associated with the variation of the distance between Mn atoms (*d*_Mn-Mn_)^20^,^21^.

Exchange bias (EB) effect, a shift of magnetization hysteresis loop along the field axis, was another interesting phenomenon in Ni-Mn-X alloys. In these alloys, Mn-Mn interaction within regular Mn sublattice is ferromagnetic (FM), while excess Mn atoms occupying Ni or X sites are antiferromagnetically coupled to Mn atoms at regular sites^22^,^23^,^24^. The resulting competing FM and antiferromagnetic (AFM) exchange interactions have been claimed to account for the EB effect in these alloys, and several different ground states have been proposed, such as superspin glass (SSG)^25^, mixed AFM/FM phases^26^, and mixed spin glass/FM phases^27^. The common feature is that an inhomogeneous ground state and the unidirectional anisotropy formed at the interface of different phases were proposed to explain the mechanism of the EB effect. In Ni-Mn-based alloys, although several efforts have been made to increase the EB field (*H*_E_), the influence of spin-orbital coupling, which has great influence on the magnetic anisotropy, have not been discussed.

In this work, we choose the substitution of Ni by Pd(Pt) in Ni_50_Mn_36_Sn_14_ alloy for the following two reasons. Since Ni, Pd, and Pt are of the same valence electron with increasing atomic radius, we can explore the influence of negative chemical pressure on the phase transitions in Ni-Mn-Sn alloys. Furthermore, we aim to study the effect of spin-orbital coupling on the unidirectional magnetic anisotropy, i.e. EB effect by introducing Pd(Pt) atoms with stronger spin-orbital coupling than Ni.

## Results and Discussions

**Figure 1:**
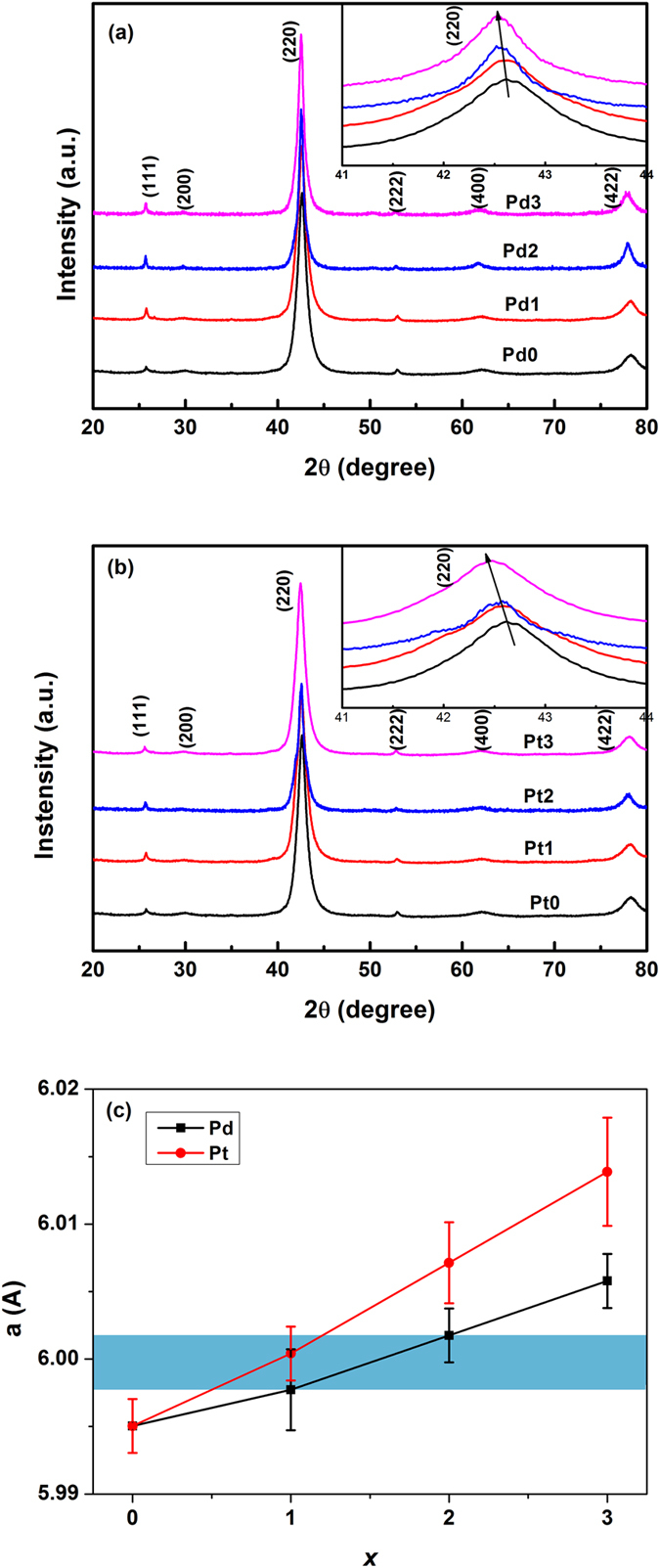
(**a**) The XRD patterns of Ni_50−x_Mn_36_Sn_14_Pd_x_ (x = 0, 1, 2, 3) alloys at room temperature; (**b**) The XRD patterns of Ni_50−x_Mn_36_Sn_14_Pt_x_ (x = 0, 1, 2, 3) alloys at room temperature; (**c**) The composition dependence of lattice constant. The blue region indicates the range of lattice constant where intermartensitic transformation appears.

Figure 1(a,b) show the XRD patterns of Ni_50−x_Mn_36_Sn_14_T_x_ (x = 0, 1, 2, 3) alloys at room temperature with T = Pd and Pt, respectively. All samples are of the pure austenitic phase with a cubic Heusler L2_1_-type structure at room temperature, indicating the MT temperature is below room temperature. In the inset of Fig. 1(a), it can be clearly seen that the (220) peaks shift towards low angles with the increasing substitutions of Ni by Pd in Ni_50_Mn_36_Sn_14_, indicating the increase of cell volume. A similar behavior was also observed in Ni_50−x_Mn_36_Sn_14_Pt_x_ (x = 0, 1, 2, 3) alloys, which could be attributed to the substitution of Pd (1.79 Å) and Pt (1.83 Å) atoms with larger radius for Ni (1.62 Å). Figure 1(c) shows the calculated lattice parameters (a) of Ni_50−x_Mn_36_Sn_14_T_x_ (T = Pd, Pt; x = 0, 1, 2, 3) alloys, and the blue region in Fig. 1(c) indicates the range of lattice parameters where IMT appears in Ni_50−x_Mn_36_Sn_14_T_x_ (T = Pd, Pt) alloys, which will be discussed in detail later. It can be seen that the value of a increases gradually with the increase of Pd(Pt) content.

**Figure 2:**
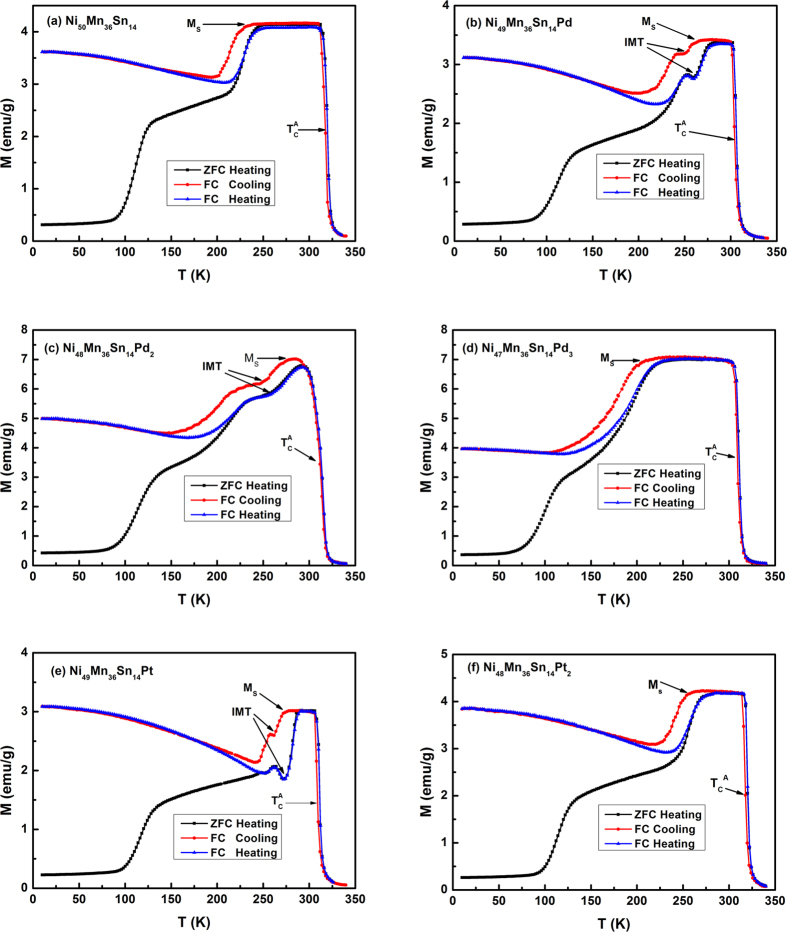
The ZFC, FCC, and FCW *M*-*T* curves in the magnetic field of 100 Oe for (**a**) Ni_50_Mn_36_Sn_14_; (**b**) Ni_49_Mn_36_Sn_14_Pd; (**c**) Ni_48_Mn_36_Sn_14_Pd_2_; (**d**) Ni_47_Mn_36_Sn_14_Pd_3_; (**e**) Ni_49_Mn_36_Sn_14_Pt; (**f**) Ni_48_Mn_36_Sn_14_Pt_2_.

It is known that in Ni-Mn-X (X = In, Sn, Sb) alloys the MT temperatures increase with the increase of e/a, which provides a convenient way to modulate the transition temperature. In the case of Ni_50−x_Mn_36_Sn_14_T_x_ alloys, since Ni, Pd, and Pt are located within the same main group, the influence of e/a can be eliminated. Figure 2 shows the temperature dependence of magnetization (*M*-*T*) for Ni_50−x_Mn_36_Sn_14_T_x_ (T = Pd, Pt) alloys. All these data were recorded upon zero field cooling (ZFC), field Cooling (FC), and field warming (FW) with an applied field of 100 Oe in the temperature range between 10 K and 340 K. Normally, the phase transitions in Ni-Mn-X alloys are characterized by the Curie temperature of austenite (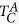), the martensitic transformation starting temperature (*M*_*s*_), and the Curie temperature of martensite (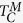). As can be seen, all curves show typical behavior with 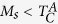 and 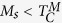. The value of *M*_s_ shows a nonmonotonous variation with the increase of Pd(Pt) content. In Ni_50−x_Mn_36_Sn_14_Pd_x_ alloys, the value of *M*_s_ increases from 231 K for x = 0 to 276 K for x = 2, and decreases to 203 K for x = 3, respectively. The variation of *M*_s_ with increasing Pd content is quite confusing since previous investigations indicate the decrease of *M*_*s*_ with the increase of cell volume in Ni-Mn-X alloys, as demonstrated by the increase of *M*_*s*_ in Ga-doped Ni-Mn-In alloy^17^, Ge-doped Ni-Mn-Sn alloy^18^, and the hydrostatic pressure effect in Ni-Mn-In alloy^12^. Recently, an increase of MT temperature was observed in Ni_2_MnGa alloy by the substitution of Pt for Ni, which has been attributed to enhanced antiferromagnetic correlations with the increase of Pt content^28^. Similar enhancement of antiferromagnetic correlations, could be expected by Pd(Pt) substitution in Ni_50−x_Mn_36_Sn_14_T_x_ (T = Pd, Pt) alloys, which will be further discussed in the composition dependence of magnetization at low temperature. Therefore, the competition of two factors may result in the nonmonotonous evolution of MT temperature with Pd(Pt) doping.

A prominent feature in Fig. 2 is the appearance of IMT below *M*_s_ for Ni_50−x_Mn_36_Sn_14_Pd_x_ (x = 1, 2) and Ni_49_Mn_36_Sn_14_Pt alloys. As can be seen in Fig. 2(b,c,e), different from *M*-*T* curves of Ni_50_Mn_36_Sn_14_ alloy [Fig. 2(a)], *M*-*T* curves of these alloys show a two-step behavior around the MT temperature. This peculiar behavior in *M*-*T* may suggest the existence of an intermartensitic phase at temperature *T*_I_ where *T*_I_ < *M*_s_, as proposed in Ni-Mn-Ga alloys^24^. Nevertheless, one may suspect that inhomogeneous phases may produce a two-step process in *M*-*T* curves considering the sensitivity of transformation temperature to composition.

**Figure 3:**
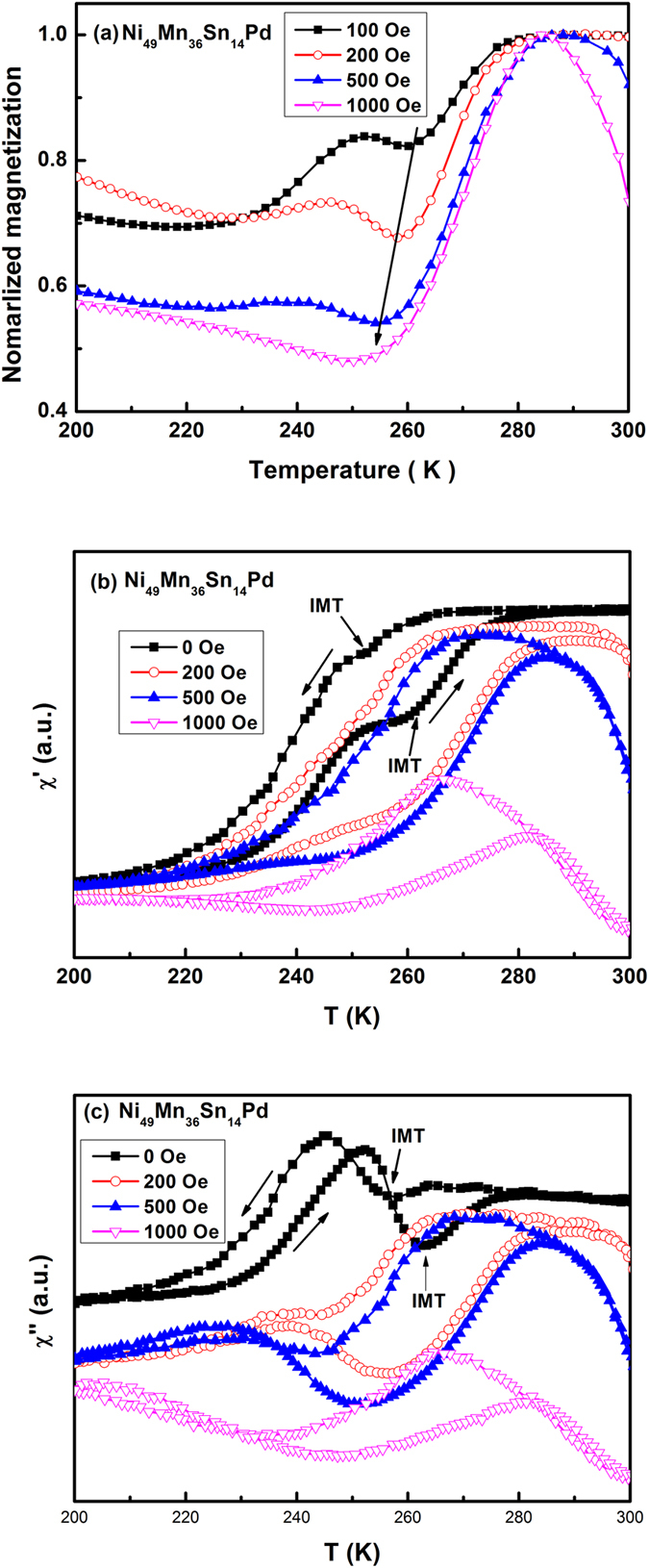
(**a**) The normalized magnetization versus temperature at the field of 100, 200, 500 and 1 kOe on heating for Ni_49_Mn_36_Sn_14_Pd alloy; (**b**) The real part of ac susceptibility at different magnetic fields; (**c**) The imaginary part of ac susceptibility at different magnetic fields.

To further investigate the two-step behavior in response to magnetic field, we have looked into the *M*-*T* curves and AC susceptibility under different magnetic field. It was found that the two-step process is highly sensitive to the magnitude of field. To demonstrate the field dependence of *M*-*T* curves more clearly, we plot the normalized magnetization versus temperature at the field of 100, 200, 500 and 1000 Oe [Fig. 3(a)] on heating for Ni_49_Mn_36_Sn_14_Pd alloy. Obviously, with the increase of applied magnetic field, the low-temperature step of transition weakened with decreased *T*_I_, and was suppressed in the field of 1 kOe. Similar behavior was also observed in Ni_48_Mn_36_Sn_14_Pd_2_ and Ni_49_Mn_36_Sn_14_Pt alloys (not shown here). Figure 3(b,c) show the real and imaginary part of ac susceptibility at different magnetic fields. Similar two-step behavior can also be observed in χ′(T) curves, and is even more distinct in χ″(T) curves. A gradual suppression of low temperature transition by magnetic field was confirmed. Since this suppression behavior should not take place in the case of transition associated with inhomogeneous phase, IMT should account for the two-step transition at low field in Ni_50−x_Mn_36_Sn_14_Pd_x_ (x = 1, 2) and Ni_49_Mn_36_Sn_14_Pt alloys.

Now let us discuss the physical mechanism for the sensitivity of IMT, i.e. the appearance and diminishment of IMT in response to the change of composition and magnetic field. In the investigation of Ni_2_MnGa single crystal, it has been shown, that the tension along the <100> direction of the ordered (L2_1_) parent phase could induce the IMT^29^. Ma *et al.* observed an IMT in high pressure annealed Ni-Co-Mn-Sn alloy, which was attributed to the enhanced magnetoelastic coupling by the application of pressure^19^. Recently, IMT was observed in Ni-Cu-Mn-Sn alloys, and it was proposed that replacing Ni for Cu generates the internal stress in the alloys, which is responsible for instability in the structure of the martensitic phase^30^. All these results indicate that the stability of martensitic phases with different structure is sensitive to the pressure (external or internal, positive or negative). Looking back to Fig. 1(c), it can be seen that IMT appears with lattice constant in a small range between 5.997 and 6.002 Å for Ni_50−x_Mn_36_Sn_14_T_x_ (T = Pd, x = 1,2; T = Pt, x = 1) alloys. Therefore, in the case of Pd(Pt) doped alloys, the small substitution of Pd(Pt) for Ni should induce proper internal tension in the crystal lattice, which makes the intermartensitic phase more stable in the corresponding temperature range. However, further increasing Pd(Pt) content makes the crystal lattice expand and suppress the intermartensitic phase, suggesting that IMT is sensitive to internal tension. The sensitivity of IMT to pressure is also demonstrated by its suppression upon the application of magnetic field in Ni_50−x_Mn_36_Sn_14_T_x_ (T = Pd, x = 1,2; T = Pt, x = 1) alloys. This phenomenon can understood by the fact that the application of magnetic field helps align the magnetic moments of the martensitic variants, which may produce internal stress in the martensitic phase and compensate the tension effect generated by Pd(Pt) doping. A similar field dependence of IMT was reported in Ni-Cu-Mn-Sn^30^ and Ni-Mn-In-Sb alloys^31^, where IMT vanished at a higher magnetic field.

**Figure 4:**
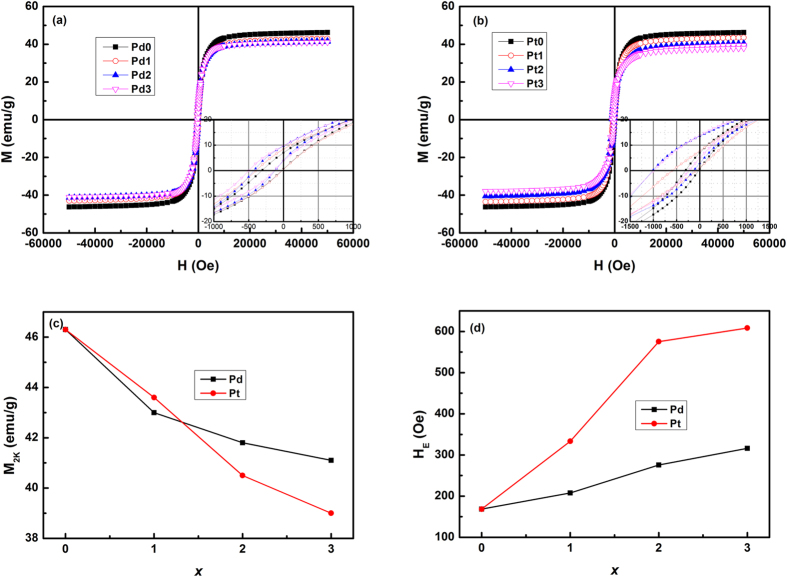
(**a**) *M*-*H* loops at 2 K after FC (*H*_FC_ = 10 kOe) from 300 K for Ni_50−x_Mn_36_Sn_14_Pd_x_ (x = 0, 1, 2, 3) alloys; (**b**) *M*-*H* loops at 2 K after FC (*H*_FC_ = 10 kOe) from 300 K for Ni_50−x_Mn_36_Sn_14_Pt_x_ (x = 0, 1, 2, 3) alloys; (**c**) The saturated magnetization at 2 K as a function of Pd(Pt) content; (**d**) *H*_E_ at 2 K after FC (*H*_FC_ = 10 kOe) from 300 K as a function of Pd(Pt) content.

At low temperature region of martensitic phase, all samples show spin-glass-like behavior characterized by the bifurcation between the FC and ZFC M(T) curves, as shown in Fig. 2. At higher temperature, however, ferromagnetic or ferrimagnetic behavior is present with Curie temperature 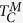 above the MT temperature, which indicates that the nature of ground state is so-called “reentrant” spin glass^32^. To further study the effect of Pd(Pt) doping on the magnetic ground state, we measured the magnetic hysteresis (*M*-*H*) loops at low temperature after field cooling (FC) in a field of 1 T from 300 K. Figure 4(a,b) show the FC *M*-*H* loops of Ni_50−x_Mn_36_Sn_14_T_x_ (x = 0, 1, 2, 3) alloys at 2 K for T = Pd and Pt, respectively. All samples exhibit the shift of *M*-*H* loops to the negative field direction, i.e. exchange bias (EB) effect, which has been observed in Ni-Mn-X (X = In, Sn, Sb) alloys and can be ascribed to the coexistence and competition of FM and AFM interaction at low temperature^25^,^26^,^33^. Recently, we proposed, due to the spatial composition fluctuation and competing FM/AFM interactions, a ground state with non-percolated FM domains in SG matrix in Ni_2_Mn_1.4_Ga_0.6_ alloy, which accounts for the appearance of zero-field exchange bias effect (ZEB)^27^. As for the case of zero field cooling process in Ni_50−x_Mn_36_Sn_14_T_x_ (x = 0, 1, 2, 3), however, *M*-*H* loops (not shown here) show no shift along the field axis, that is, no zero-field exchange bias effect was observed in the Ni_50−x_Mn_36_Sn_14_T_x_ (T = Pd, Pt) alloys. Combined with the relative large value of magnetization at low temperature, we suggest that the possible ground state can be percolated FM region coexisting with SG phase, and this can result in the formation of unidirectional exchange anisotropy at the interface between FM and SG phases upon FC process.

Figure 4(c) shows the composition dependences of saturated magnetization (M_2K_) for Ni_50−x_Mn_36_Sn_14_T_x_ (T = Pd, Pt) alloys at 2 K. Clearly, the magnetization decreases gradually with the increasing Pd(Pt) content, suggesting the decrease of FM proportion in the mixed phases. From FC *M-H* loops in Fig. 4(a,b), the exchange bias field (*H*_E_) and coercivity (*H*c) are determined as *H*_E_  = −(*H*_1_ + *H*_2_)/2 and *H*_C_  = −(*H*_1_ − *H*_2_)/2, respectively, where *H*_1_ and *H*_2_ are the left and right coercive fields. Figure 4(d) shows *H*_*E*_ at 2 K after FC (*H*_*FC*_ = 10 kOe) from 300 K as a function of Pd(Pt) content. It was found that *H*_E_ increases notably with increasing amount of Pd(Pt) doping: *H*_E_ increases from 168 Oe for x = 0 to 316 Oe for x = 3 in Ni_50−x_Mn_36_Sn_14_Pd_x_ alloy, and increases from 168 Oe for x = 0 to 609 Oe for x = 3 in Ni_50−x_Mn_36_Sn_14_Pt_x_. The distinct increase of *H*_E_ by Pd(Pt) addition can be considered from the following two aspects. One is the variation of FM/SG phase ratio. It has been reported in FM/AFM bilayer, *H*_E_ can be described as *H*_E_ = *J*_int_/(*M*_FM_·t_FM_), where *J*_int_ is the interface coupling constant, *M*_FM_ is the saturation magnetization of FM layer, and *t*_FM_ is the thickness of FM layer^34^. Similarly, for the case of Ni_50−x_Mn_36_Sn_14_T_x_ (T = Pd, Pt) alloys, (*M*_FM_·*t*_FM_) can be regarded as the fraction of FM phase, and *J*_int_ represents the mean exchange energy at the FM/SG interface. With the substitution of Ni by Pd(Pt), the FM fraction decreases as demonstrated by the decrease of magnetization, thus leading to enhancement of *H*_E_. This mechanism can explain the magnetization dependence of *H*_E_ in Ni-Mn-Sb^33^ and Ni-Mn-Sn alloys^35^. Nevertheless, as the change of magnetization is relatively small, we believe this factor should work but is not the dominant reason for the increase of *H*_E_. The other reason may be associated with the stronger spin-orbital coupling of Pd(Pt) atom than that of Ni, which gives rise to magnetic anisotropy and consequently increases the value of *H*_E_. It has been reported in canonical CuMn SG system, the introduction of nonmagnetic Au(Pt) impurities with strong spin-orbit coupling can largely enhance the magnetic anisotropy^36^. This has been attributed to an additional term in the Ruderman-Kittel-Kasuya-Yosida (RKKY) interaction which is of the Dzyaloshinsky-Moriya (DM) type and is due to spin-orbit scattering of the conduction electrons by the nonmagnetic impurities^37^,^38^. Similarly, in Ni_50−x_Mn_36_Sn_14_T_x_ (T = Pd, Pt) alloys, Pd(Pt) doping may also increase the unidirectional anisotropy of SG phase due to DM interaction between the Mn spins, and subsequently lead to the increase of *H*_E_. This could also explain why the addition of Pt increases *H*_E_ more sharply than that of Pd by the fact that the strength of spin-orbital coupling follows Pt > Pd > Ni. Recently, Nayak *et al.* obtained a giant EB of more than 3 T in the vicinity of the compensation composition in Mn–Pt–Ga system^39^. The large exchange anisotropy has been attributed to the exchange interaction between the compensated host and ferrimagnetic clusters due to intrinsic anti-site disorder. We believe that the effect of strong spin orbital coupling, although not discussed by the authors, should play an important role in the giant EB of Mn–Pt–Ga alloy, considering that the value of *H*_E_ in Mn–Pt–Ga is much larger that in Mn-Fe-Ga. These results suggest that introducing the elements with strong spin-orbit coupling may provide a general way to enhance the EB effect in Heusler alloys.

**Figure 5:**
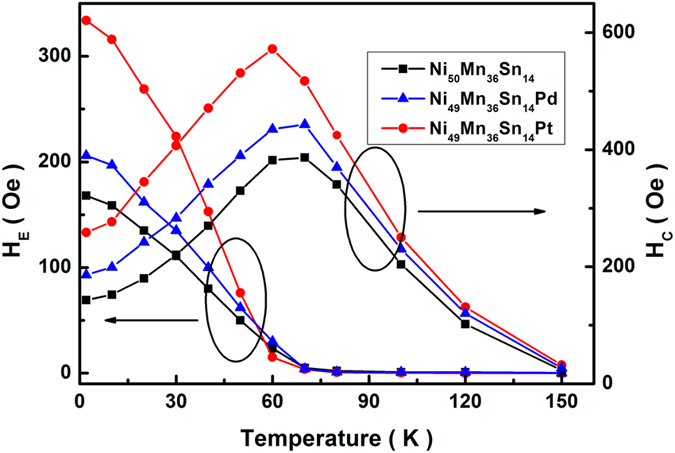
The temperature dependence of *H*_E_ and *H*_C_ for Ni_49_Mn_36_Sn_14_T (T = Ni, Pd, Pt) alloys after FC (*H*_FC_ = 10 kOe) from 300 K.

Figure 5 shows the temperature dependence of *H*_E_ and *H*_C_ for Ni_49_Mn_36_Sn_14_T (T = Ni, Pd, Pt) alloys after FC (*H*_*FC*_ = 10 kOe) from 300 K. It can be seen that all alloys show similar temperature dependence of *H*_E_ and *H*_C_: the values of *H*_E_ decrease almost linearly with increasing temperature and become zero around the blocking temperature (*T*_B_ = 70 K), where the values of *H*_C_ reach the maximum value. The similar phenomenon was also found in Co(FM)/CuMn(SG) bilayer as well as convention FM/AFM systems due to the decrease of SG (or AFM) anisotropy close to *T*_B_^34^,^40^. In Ni_49_Mn_36_Sn_14_T (T = Ni, Pd, Pt) alloys, the magnetic anisotropy of SG phase (*K*_SG_) decreases with the increasing temperature, which makes FM phase can drag more SG spins, causing the increase in *H*_C_; until at *T*_*B*_, SG spins can no longer hinder the FM rotation and consequently *H*_*E*_ becomes zero.

In summary, we have investigated the effects of Pd(Pt) substitution for Ni on the crystal structure, phase transitions and EB effect in Ni_50−x_Mn_36_Sn_14_T_x_ (T = Ni, Pd, Pt) Heusler alloys. With the increase of Pd(Pt) content, the lattice parameter increases gradually, while the MT temperature shows nonmonotonous composition dependence. The appearance of IMT was observed by small Pd(Pt) addition in Ni_50−x_Mn_36_Sn_14_T_x_ with x = 1, 2 for T = Pd and x = 1 for T = Pt, and it can be suppressed by the application of magnetic field as well as further Pd(Pt) doping. These results indicate that IMT in Ni_50−x_Mn_36_Sn_14_T_x_ alloys is highly sensitive to pressure, such as chemical pressure by doping and internal stress by magnetic field. All samples exhibit a “reentrant” spin glass behavior at low temperature, and a significant enhancement of EB effect after FC treatments was obtained by Pd(Pt) doping. EB effect has been explained in terms of coexistence of percolated FM region and SG phase. The decreased FM proportion and Dzyaloshinsky-Moriya interactions in the SG phase may account for the increase of *H*_*E*_. The latter mechanism plays an important role and provides an effective way to improve the EB effect in Heusler alloys.

## Methods

Ni_50−x_Mn_36_Sn_14_T_x_ (T = Pd, Pt; x = 0, 1, 2, 3) polycrystalline alloys were prepared by arc melting the appropriate amounts of Ni, Mn, Sn, Pd, Pt in argon atmosphere. These alloys were sealed in quartz tubes and annealed at 1173 K for 72 h followed by quenching in water. The crystal structures were identified by the X-ray diffraction (XRD) using Cu-Kα radiation at room temperature. Magnetic measurements were carried out using a physical property measurement system (PPMS, Quantum Design Evercool-2).

## Additional Information

**How to cite this article**: Dong, S. Y. *et al.* Intermartensitic Transformation and Enhanced Exchange Bias in Pd (Pt) -doped Ni-Mn-Sn alloys. *Sci. Rep.* **6**, 25911; doi: 10.1038/srep25911 (2016).
